# 9-*p*-Tolyl-9*H*-carbazole-3-carbonitrile

**DOI:** 10.1107/S1600536811039286

**Published:** 2011-09-30

**Authors:** C. Ramathilagam, N. Venkatesan, P. Rajakumar, P. R. Umarani, V. Manivannan

**Affiliations:** a Department of Physics, AMET University, Kanathur, Chennai 603 112, India; b Department of Organic Chemistry, University of Madras, Guindy campus, Chennai 600 025, India; c Department of Physics, Presidency College (Autonomous), Chennai 600 005, India; d Department of Research and Development, PRIST University, Vallam, Thanjavur 613 403, Tamil Nadu, India

## Abstract

In the title compound, C_20_H_14_N_2_, the carbazole ring system is essentially planar (r.m.s. deviation = 0.187 Å) and is inclined at an angle of 54.33 (4) ° with respect to the benzene ring. The crystal packing is stabilized by weak C—H⋯N and C—H⋯π inter­actions.

## Related literature

For the biological activity of carbazole derivatives, see: Ramsewak *et al.* (1999 ▶); Tachibana *et al.* (2001 ▶); Itoigawa *et al.* (2000 ▶). For related structures, see: Archana *et al.* (2010 ▶); Velmurugan *et al.* (2010 ▶); Yuan *et al.* (2010 ▶).
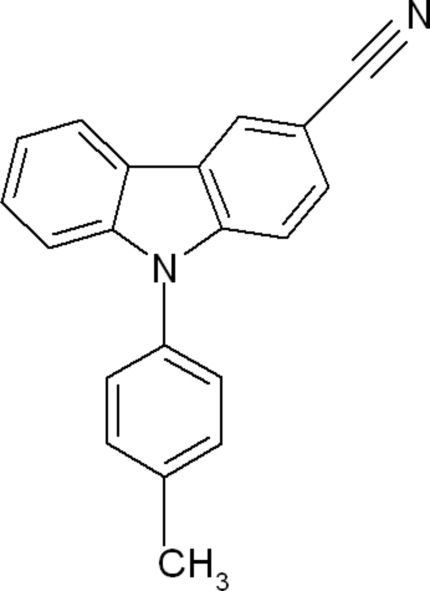


## Experimental

### 

#### Crystal data

C_20_H_14_N_2_
*M* *_r_* = 282.33
Triclinic, 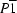

*a* = 8.6031 (3) Å
*b* = 8.8247 (3) Å
*c* = 10.4609 (4) Å
α = 80.514 (2)°
β = 87.499 (2)°
γ = 72.114 (2)°
*V* = 745.45 (5) Å^3^
*Z* = 2
Mo *K*α radiation
μ = 0.08 mm^−1^
*T* = 295 K
0.22 × 0.19 × 0.17 mm

#### Data collection

Bruker Kappa APEXII CCD diffractometer
Absorption correction: multi-scan (*SADABS*; Sheldrick, 1996 ▶) *T*_min_ = 0.984, *T*_max_ = 0.987
13631 measured reflections
3724 independent reflections
2695 reflections with *I* > 2σ(*I*)
*R*_int_ = 0.029

#### Refinement

*R*[*F* ^2^ > 2σ(*F* ^2^)] = 0.043
*wR*(*F* ^2^) = 0.123
*S* = 1.04
3724 reflections
200 parameters
1 restraint
H-atom parameters constrained
Δρ_max_ = 0.16 e Å^−3^
Δρ_min_ = −0.21 e Å^−3^

### 

Data collection: *APEX2* (Bruker, 2004 ▶); cell refinement: *SAINT* (Bruker, 2004 ▶); data reduction: *SAINT*; program(s) used to solve structure: *SHELXS97* (Sheldrick, 2008 ▶); program(s) used to refine structure: *SHELXL97* (Sheldrick, 2008 ▶); molecular graphics: *PLATON* (Spek, 2009 ▶); software used to prepare material for publication: *SHELXL97*.

## Supplementary Material

Crystal structure: contains datablock(s) global, I. DOI: 10.1107/S1600536811039286/pv2449sup1.cif

Structure factors: contains datablock(s) I. DOI: 10.1107/S1600536811039286/pv2449Isup2.hkl

Supplementary material file. DOI: 10.1107/S1600536811039286/pv2449Isup3.cml

Additional supplementary materials:  crystallographic information; 3D view; checkCIF report

## Figures and Tables

**Table 1:** Hydrogen-bond geometry (Å, °) *Cg*2 is the centroid of the C1–C6 ring.

*D*—H⋯*A*	*D*—H	H⋯*A*	*D*⋯*A*	*D*—H⋯*A*
C8—H8⋯N2^i^	0.93	2.57	3.434 (2)	154
C15—H15⋯*Cg*2^ii^	0.93	2.71	3.453 (1)	137

Symmetry codes: (i) 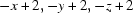; (ii) 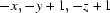.

**Fig. 1.**
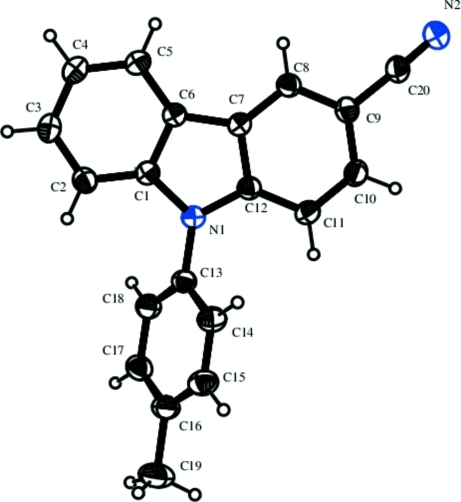
The molecular structure of the title compound with 30% probability displacement ellipsoids for the non-H atoms.

## supplementary crystallographic information

### Comment

Carbazole derivatives possess antioxidative (Tachibana *et al.*, 2001), antitumor (Itoigawa *et al.*, 2000), anti-inflammatory and antimutagenic (Ramsewak *et al.*, 1999) activities.

The geometric parameters of the title molecule (Fig. 1) agree well with the corresponding geometric parameters reported in similar structures (Archana *et al.*, 2010; Velmurugan *et al.*, 2010; Yuan *et al.*, 2010). The carbazole ring system is essentially planar with maximum deviation of C9 from the least-squae plane defined by the atoms N1/C1–C12 being 0.0306 (10) Å. The mean plane of the carbazole ring system makes a dihedral angle of 54.33 (4) ° with the phenyl ring. The sum of bond angles around N1 [359.87 (10) °]indicates the *sp^2^* hybridization state of atom N1 in the molecule. The crystal packing of the compound is stabilized by weak C8—H8···N2 hydrogen bonds and C15—H15···π interactions involving the centroid of C1–C6 ring (Table 1).

### Experimental

To a stirred solution of AlCl_3_ (2.8 g, 2.1 mmol), in dry THF (100 ml) sodium azide (4.1 g, 6.31 mmol), and 9-*p*-tolyl-9*H*-carbazole-3-carbaldehyde (3 g, 1.05 mmol) were added and the resulting mixture was heated to gentle reflux. The progress of the reaction was monitored by TLC. The suspension gradually turned pale yellow after 5–6 h. Then excess THF was removed by distillation and the residue was diluted with 10% HCl (10 ml). The aqueous layer was extracted with CHCl_3_ (2x50 ml) and brine (25 ml). The organic layer was separated and dried over anhydrous sodium sulfate. The solvent was distilled off under reduced pressure and the residue was purified by column chromatography by elution with mixture of ethyl acetate and hexane (1:4) to give the title compound as colorless crystalline solid.(m.p 449 K).

### Refinement

The H atoms were positioned geometrically and refined using riding model with C—H = 0.93 and 0.96Å for aryl and methyl type H-atoms, respectively, and *U*_iso_(H) = 1.2 or 1.5 times *U*_eq_(C) for aromatic or methyl H-atoms.

### Crystal data

**Table:**

C_20_H_14_N_2_	*Z* = 2
*M**_r_* = 282.33	*F*(000) = 296
Triclinic, *P*1	*D*_x_ = 1.258 Mg m^−^^3^
Hall symbol: -P 1	Mo *K*α radiation, λ = 0.71073 Å
*a* = 8.6031 (3) Å	Cell parameters from 3724 reflections
*b* = 8.8247 (3) Å	θ = 2.0–28.4°
*c* = 10.4609 (4) Å	µ = 0.08 mm^−^^1^
α = 80.514 (2)°	*T* = 295 K
β = 87.499 (2)°	Block, colourless
γ = 72.114 (2)°	0.22 × 0.19 × 0.17 mm
*V* = 745.45 (5) Å^3^	

### Data collection

**Table:**

Bruker Kappa APEXII CCD diffractometer	3724 independent reflections
Radiation source: fine-focus sealed tube	2695 reflections with *I* > 2σ(*I*)
graphite	*R*_int_ = 0.029
Detector resolution: 0 pixels mm^-1^	θ_max_ = 28.4°, θ_min_ = 2.0°
ω and φ scans	*h* = −11→9
Absorption correction: multi-scan (*SADABS*; Sheldrick, 1996)	*k* = −11→11
*T*_min_ = 0.984, *T*_max_ = 0.987	*l* = −13→13
13631 measured reflections	

### Refinement

**Table:**

Refinement on *F*^2^	Primary atom site location: structure-invariant direct methods
Least-squares matrix: full	Secondary atom site location: difference Fourier map
*R*[*F*^2^ > 2σ(*F*^2^)] = 0.043	Hydrogen site location: inferred from neighbouring sites
*wR*(*F*^2^) = 0.123	H-atom parameters constrained
*S* = 1.04	*w* = 1/[σ^2^(*F*_o_^2^) + (0.0561*P*)^2^ + 0.1087*P*] where *P* = (*F*_o_^2^ + 2*F*_c_^2^)/3
3724 reflections	(Δ/σ)_max_ = 0.002
200 parameters	Δρ_max_ = 0.16 e Å^−^^3^
1 restraint	Δρ_min_ = −0.21 e Å^−^^3^

### Special details

**Table:**

Geometry. All e.s.d.'s (except the e.s.d. in the dihedral angle between two l.s. planes) are estimated using the full covariance matrix. The cell e.s.d.'s are taken into account individually in the estimation of e.s.d.'s in distances, angles and torsion angles; correlations between e.s.d.'s in cell parameters are only used when they are defined by crystal symmetry. An approximate (isotropic) treatment of cell e.s.d.'s is used for estimating e.s.d.'s involving l.s. planes.

### Fractional atomic coordinates and isotropic or equivalent isotropic displacement parameters (Å^2^)

**Table:**

	*x*	*y*	*z*	*U*_iso_*/*U*_eq_	
C1	1.15482 (15)	0.39757 (15)	0.76616 (11)	0.0428 (3)	
C2	1.28283 (17)	0.27239 (16)	0.72999 (13)	0.0503 (3)	
H2	1.2641	0.1969	0.6851	0.060*	
C3	1.43894 (17)	0.26447 (17)	0.76330 (13)	0.0544 (3)	
H3	1.5267	0.1814	0.7408	0.065*	
C4	1.46875 (17)	0.37727 (17)	0.82957 (13)	0.0540 (3)	
H4	1.5755	0.3684	0.8503	0.065*	
C5	1.34207 (15)	0.50179 (16)	0.86478 (12)	0.0482 (3)	
H5	1.3622	0.5774	0.9087	0.058*	
C6	1.18294 (15)	0.51223 (14)	0.83321 (11)	0.0413 (3)	
C7	1.02493 (14)	0.62392 (14)	0.85320 (11)	0.0408 (3)	
C8	0.97472 (15)	0.75589 (15)	0.91712 (12)	0.0447 (3)	
H8	1.0505	0.7899	0.9561	0.054*	
C9	0.80848 (16)	0.83680 (15)	0.92188 (12)	0.0470 (3)	
C10	0.69339 (16)	0.78683 (16)	0.86297 (13)	0.0513 (3)	
H10	0.5829	0.8431	0.8670	0.062*	
C11	0.74155 (16)	0.65580 (16)	0.79929 (13)	0.0497 (3)	
H11	0.6653	0.6228	0.7599	0.060*	
C12	0.90815 (15)	0.57363 (15)	0.79534 (11)	0.0428 (3)	
C13	0.91238 (15)	0.35356 (16)	0.67085 (12)	0.0447 (3)	
C14	0.81166 (17)	0.43835 (18)	0.56626 (12)	0.0531 (3)	
H14	0.7938	0.5487	0.5431	0.064*	
C15	0.73821 (18)	0.3594 (2)	0.49679 (13)	0.0588 (4)	
H15	0.6688	0.4181	0.4282	0.071*	
C16	0.76514 (18)	0.1943 (2)	0.52662 (14)	0.0582 (4)	
C17	0.86723 (18)	0.11099 (18)	0.63019 (15)	0.0582 (4)	
H17	0.8883	−0.0001	0.6512	0.070*	
C18	0.93897 (17)	0.18914 (16)	0.70359 (13)	0.0517 (3)	
H18	1.0047	0.1313	0.7745	0.062*	
C19	0.6847 (3)	0.1091 (3)	0.4495 (2)	0.0915 (6)	
H19A	0.5682	0.1520	0.4557	0.137*	
H19B	0.7182	−0.0041	0.4832	0.137*	
H19C	0.7163	0.1249	0.3603	0.137*	
C20	0.75645 (17)	0.97199 (16)	0.99028 (14)	0.0537 (3)	
N1	0.98705 (12)	0.43589 (13)	0.74328 (10)	0.0454 (3)	
N2	0.72084 (16)	1.07784 (16)	1.04614 (14)	0.0700 (4)	

### Atomic displacement parameters (Å^2^)

**Table:**

	*U*^11^	*U*^22^	*U*^33^	*U*^12^	*U*^13^	*U*^23^
C1	0.0421 (6)	0.0455 (7)	0.0405 (6)	−0.0140 (5)	0.0003 (5)	−0.0053 (5)
C2	0.0519 (8)	0.0486 (7)	0.0508 (7)	−0.0142 (6)	0.0048 (6)	−0.0122 (6)
C3	0.0440 (7)	0.0523 (8)	0.0608 (8)	−0.0070 (6)	0.0063 (6)	−0.0083 (6)
C4	0.0411 (7)	0.0592 (8)	0.0590 (8)	−0.0140 (6)	−0.0018 (6)	−0.0039 (6)
C5	0.0438 (7)	0.0531 (7)	0.0482 (7)	−0.0157 (6)	−0.0035 (5)	−0.0061 (6)
C6	0.0420 (6)	0.0432 (6)	0.0386 (6)	−0.0137 (5)	0.0001 (5)	−0.0049 (5)
C7	0.0397 (6)	0.0426 (6)	0.0398 (6)	−0.0132 (5)	−0.0003 (5)	−0.0044 (5)
C8	0.0462 (7)	0.0432 (7)	0.0461 (6)	−0.0154 (5)	−0.0011 (5)	−0.0072 (5)
C9	0.0486 (7)	0.0412 (6)	0.0489 (7)	−0.0112 (5)	0.0020 (5)	−0.0060 (5)
C10	0.0409 (7)	0.0484 (7)	0.0616 (8)	−0.0097 (5)	0.0015 (6)	−0.0079 (6)
C11	0.0413 (7)	0.0525 (7)	0.0572 (7)	−0.0163 (6)	−0.0021 (6)	−0.0096 (6)
C12	0.0435 (7)	0.0425 (6)	0.0432 (6)	−0.0143 (5)	−0.0006 (5)	−0.0061 (5)
C13	0.0451 (7)	0.0519 (7)	0.0417 (6)	−0.0197 (6)	0.0029 (5)	−0.0120 (5)
C14	0.0586 (8)	0.0568 (8)	0.0467 (7)	−0.0234 (7)	−0.0034 (6)	−0.0040 (6)
C15	0.0592 (9)	0.0763 (10)	0.0456 (7)	−0.0267 (8)	−0.0038 (6)	−0.0098 (7)
C16	0.0549 (8)	0.0776 (10)	0.0552 (8)	−0.0319 (7)	0.0074 (6)	−0.0261 (7)
C17	0.0610 (9)	0.0531 (8)	0.0678 (9)	−0.0241 (7)	0.0063 (7)	−0.0184 (7)
C18	0.0529 (8)	0.0509 (7)	0.0524 (7)	−0.0168 (6)	−0.0016 (6)	−0.0085 (6)
C19	0.0912 (14)	0.1118 (16)	0.0975 (14)	−0.0513 (12)	−0.0035 (11)	−0.0491 (12)
C20	0.0492 (7)	0.0454 (7)	0.0637 (8)	−0.0097 (6)	0.0016 (6)	−0.0108 (6)
N1	0.0418 (6)	0.0472 (6)	0.0496 (6)	−0.0141 (5)	−0.0016 (4)	−0.0133 (5)
N2	0.0611 (8)	0.0583 (8)	0.0926 (10)	−0.0125 (6)	−0.0004 (7)	−0.0279 (7)

### Geometric parameters (Å, °)

**Table:**

C1—C2	1.3894 (18)	C11—C12	1.3958 (18)
C1—N1	1.3995 (16)	C11—H11	0.9300
C1—C6	1.4048 (17)	C12—N1	1.3834 (16)
C2—C3	1.380 (2)	C13—C18	1.3835 (18)
C2—H2	0.9300	C13—C14	1.3865 (18)
C3—C4	1.392 (2)	C13—N1	1.4230 (16)
C3—H3	0.9300	C14—C15	1.3750 (19)
C4—C5	1.3761 (19)	C14—H14	0.9300
C4—H4	0.9300	C15—C16	1.386 (2)
C5—C6	1.3942 (17)	C15—H15	0.9300
C5—H5	0.9300	C16—C17	1.382 (2)
C6—C7	1.4434 (17)	C16—C19	1.505 (2)
C7—C8	1.3829 (17)	C17—C18	1.3869 (19)
C7—C12	1.4100 (17)	C17—H17	0.9300
C8—C9	1.3914 (18)	C18—H18	0.9300
C8—H8	0.9300	C19—H19A	0.9600
C9—C10	1.4021 (19)	C19—H19B	0.9600
C9—C20	1.4356 (18)	C19—H19C	0.9600
C10—C11	1.3740 (18)	C20—N2	1.1386 (17)
C10—H10	0.9300		
			
C2—C1—N1	129.38 (12)	C12—C11—H11	120.9
C2—C1—C6	121.43 (12)	N1—C12—C11	129.52 (12)
N1—C1—C6	109.18 (11)	N1—C12—C7	109.05 (11)
C3—C2—C1	117.33 (13)	C11—C12—C7	121.40 (12)
C3—C2—H2	121.3	C18—C13—C14	119.34 (12)
C1—C2—H2	121.3	C18—C13—N1	120.73 (11)
C2—C3—C4	121.90 (13)	C14—C13—N1	119.93 (12)
C2—C3—H3	119.0	C15—C14—C13	120.07 (13)
C4—C3—H3	119.0	C15—C14—H14	120.0
C5—C4—C3	120.78 (13)	C13—C14—H14	120.0
C5—C4—H4	119.6	C14—C15—C16	121.56 (14)
C3—C4—H4	119.6	C14—C15—H15	119.2
C4—C5—C6	118.60 (13)	C16—C15—H15	119.2
C4—C5—H5	120.7	C17—C16—C15	117.77 (13)
C6—C5—H5	120.7	C17—C16—C19	121.14 (16)
C5—C6—C1	119.96 (12)	C15—C16—C19	121.09 (15)
C5—C6—C7	133.56 (12)	C16—C17—C18	121.52 (14)
C1—C6—C7	106.48 (11)	C16—C17—H17	119.2
C8—C7—C12	119.87 (11)	C18—C17—H17	119.2
C8—C7—C6	133.07 (11)	C13—C18—C17	119.71 (13)
C12—C7—C6	107.03 (10)	C13—C18—H18	120.1
C7—C8—C9	118.63 (11)	C17—C18—H18	120.1
C7—C8—H8	120.7	C16—C19—H19A	109.5
C9—C8—H8	120.7	C16—C19—H19B	109.5
C8—C9—C10	121.09 (12)	H19A—C19—H19B	109.5
C8—C9—C20	118.52 (12)	C16—C19—H19C	109.5
C10—C9—C20	120.38 (12)	H19A—C19—H19C	109.5
C11—C10—C9	120.90 (12)	H19B—C19—H19C	109.5
C11—C10—H10	119.6	N2—C20—C9	177.51 (16)
C9—C10—H10	119.6	C12—N1—C1	108.25 (10)
C10—C11—C12	118.11 (12)	C12—N1—C13	126.03 (10)
C10—C11—H11	120.9	C1—N1—C13	125.59 (10)
			
N1—C1—C2—C3	178.70 (12)	C8—C7—C12—C11	0.94 (18)
C6—C1—C2—C3	0.40 (19)	C6—C7—C12—C11	179.09 (11)
C1—C2—C3—C4	−0.5 (2)	C18—C13—C14—C15	0.5 (2)
C2—C3—C4—C5	0.1 (2)	N1—C13—C14—C15	−179.43 (12)
C3—C4—C5—C6	0.3 (2)	C13—C14—C15—C16	−1.6 (2)
C4—C5—C6—C1	−0.38 (18)	C14—C15—C16—C17	0.8 (2)
C4—C5—C6—C7	−179.43 (12)	C14—C15—C16—C19	−179.72 (14)
C2—C1—C6—C5	0.02 (18)	C15—C16—C17—C18	0.9 (2)
N1—C1—C6—C5	−178.58 (11)	C19—C16—C17—C18	−178.53 (14)
C2—C1—C6—C7	179.31 (11)	C14—C13—C18—C17	1.2 (2)
N1—C1—C6—C7	0.70 (13)	N1—C13—C18—C17	−178.86 (12)
C5—C6—C7—C8	−4.1 (2)	C16—C17—C18—C13	−1.9 (2)
C1—C6—C7—C8	176.79 (13)	C8—C9—C20—N2	−8(4)
C5—C6—C7—C12	178.13 (13)	C10—C9—C20—N2	171 (4)
C1—C6—C7—C12	−1.01 (13)	C11—C12—N1—C1	−178.47 (12)
C12—C7—C8—C9	−0.38 (17)	C7—C12—N1—C1	−0.55 (13)
C6—C7—C8—C9	−177.97 (12)	C11—C12—N1—C13	5.6 (2)
C7—C8—C9—C10	−0.23 (19)	C7—C12—N1—C13	−176.45 (11)
C7—C8—C9—C20	178.90 (11)	C2—C1—N1—C12	−178.57 (12)
C8—C9—C10—C11	0.3 (2)	C6—C1—N1—C12	−0.11 (13)
C20—C9—C10—C11	−178.79 (12)	C2—C1—N1—C13	−2.6 (2)
C9—C10—C11—C12	0.22 (19)	C6—C1—N1—C13	175.81 (11)
C10—C11—C12—N1	176.86 (12)	C18—C13—N1—C12	−128.91 (14)
C10—C11—C12—C7	−0.84 (19)	C14—C13—N1—C12	51.05 (17)
C8—C7—C12—N1	−177.18 (11)	C18—C13—N1—C1	55.88 (17)
C6—C7—C12—N1	0.97 (13)	C14—C13—N1—C1	−124.16 (14)

### Hydrogen-bond geometry (Å, °)

**Table:**

Cg2 is the centroid of the C1–C6 ring.

**Table:**

*D*—H···*A*	*D*—H	H···*A*	*D*···*A*	*D*—H···*A*
C8—H8···N2^i^	0.93	2.57	3.434 (2)	154
C15—H15···Cg2^ii^	0.93	2.71	3.453 (1)	137

Symmetry codes: (i) −*x*+2, −*y*+2, −*z*+2; (ii) −*x*, −*y*+1, −*z*+1.
